# High-resolution acoustic surveys with diving gliders come at a cost of aliasing moving targets

**DOI:** 10.1371/journal.pone.0201816

**Published:** 2018-08-16

**Authors:** Damien Guihen

**Affiliations:** Australian Maritime College, University of Tasmania, Newnham, Tasmania, Australia; University of Waikato, NEW ZEALAND

## Abstract

Underwater gliders are autonomous robots that follow a slow, see-saw path and may be deployed for months on end. Gliders have a dramatically lower payload capacity than research vessels and are thus limited to more simple instrumentation. They have the advantage, however, of being deployable for long periods of time without the high running costs of a ship. Recent years have seen development of the use of gliders to undertake acoustic surveys of biomass in the pelagic environment, highlighting their potential to fill future survey gaps. Here it is shown, using simulation of sampling, that gliders can resolve acoustic targets at greater resolutions than ships, due to their diving pattern, but that survey accuracy is strongly dependent on the speed of the target.

## Introduction

Underwater gliders are a class of Autonomous Underwater Vehicles (AUV), are self-contained, battery powered vehicles and use small changes in their buoyancy to affect a vertical movement. Pitching back and forth through the movement of an internal mass enables wings attached to the body to convert a portion of the vertical velocity to horizontal travel[1,2]. Gliders therefore travel in a highly energy-efficient seesaw pattern, as deep as 1000 m, with deeper models in development [3]. A glider will cover approximately 20 km in one day, travelling at a typical speed of 0.25 ms^-1^, while diving to depths of 1000 m. The efficient motion and limited power budget of a glider allows for deployments that may have an endurance of months. Gliders with a low-power, integrated and calibrated echo-sounder already been deployed to make measurements of acoustic backscatter[4,5].

Gliders have four fundamental differences from traditional research vessels;

1. Gliders are commanded remotely between dives, rather than having a person in the loop
2. Gliders carry much smaller payloads with a fraction of the power budget of ships
3. Gliders do not operate at a fixed depth, diving through the water column
4. Gliders move slowly: approximately 0.25 ms^-1^ [1], 1/20th the horizontal velocity of a typical ship transect [6].

A ship-based echo-sounder is typically fixed at a depth near the surface, and as such the data collected at each acoustic range is a function of water depth. The diving of the glider effectively decouples the range of the acoustic backscatter from the water depth and so even targets that are beyond the range of surface vessels are potentially detectable in the near-ranges of a glider’s acoustic dataset. An additional benefit of the glider is the ability to make measurements of temperature, salinity, dissolved oxygen concentration and chlorophyll fluorescence from very close to the insonified volumes. Towed acoustic systems can provide acoustic backscatter data from depths hundreds of meters below the range of a ship-based echo-sounder, and at closer range to deep targets, significantly reducing the range effects of acoustic observation[7], though such systems still rely on the presence of a research vessel and the regions with complex terrain may be unsuitable for towed systems. Acoustic systems have been integrated into propeller-driven AUVs since the late 1990s[8], and used to explore pelagic distributions in regions inaccessible to ships [9], to make observations of behavior independently of ships [10,11]and to explore these relationships at different depths of the ocean [12]. These torpedo-shaped AUVs have great potential to observe processes in the ocean but their power demands, typically vastly in excess of those gliders, currently limit their endurance to several days, though long-endurance variants are in development [13].

Gliders and torpedo-style AUVs therefore occupy different sensing niches, with gliders limited to relatively simple acoustic instruments [4], deployable for durations of several months. Multifrequency glider-borne echo-sounders are in development but field trials have not been published to date. AUVs can carry payloads that are comparable with those on research vessels, including the larger transducers required for lower frequency studies [12], allowing greater detail and target discrimination, but for durations measured in days. AUVs have the additional benefit of optional high-quality inertial navigation systems [14], while gliders have a much less precise flight control system and navigational suite. In general terms, AUVs are best suited for targeted process studies and mapping surveys and are a particular boon for areas inaccessible to ships, such as under ice [15] and the deep ocean. Gliders on the other hand are best suited for larger, synoptic scale surveys, particularly as a component of a multi-node observing system [16,17]. The Southern Ocean Observing System report Seeing Below The Ice [18] gives a thorough overview of the different roles and zones in Antarctic oceanography particularly in need of the different platforms, which are generally transferable to different regions around the world. For point-based samples, such as temperature and chlorophyll fluorescence, the data provided by a glider is similar in many ways to that of an Argo float [19], and the two platforms share a common ancestor in the SOFAR float [1], the divergence being the addition of hydrodynamic wings and steering to the gliders to allow for directed profiling and transecting. Beam projection from gliders, such as with echo-sounders, introduces a novel perspective on collected data as the instrument’s horizontal motion covaries with depth.

**Fig 1:**
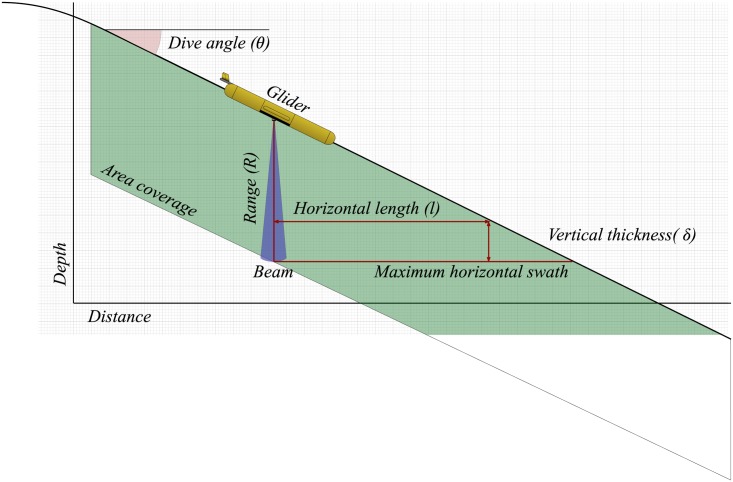
Diagram of a glider dive and acoustic coverage area with a downward-looking. The relationship between the beam, dive angle and coverage dimensions is described in Eq 1.

The see-saw path of the glider has the consequence of reducing effective horizontal acoustic coverage of a downward-facing echo-sounder. A ship may steam in a straight line, horizontal coverage will be consistent and the maximum resolving length of a large target will be limited only by the length of the ship’s transect. Acoustic measurements made from ships have been used to identify swarms of Antarctic krill (*Euphausia superba*) that were kilometers long[6]. A glider’s path will take it through insonified depths, thus it will intersect its swath at a particular depth, limiting its horizontal extent. A simple trigonometric arrangement shows the maximum horizontal length, *l*, for an echo-rounder of range *R*, vertical thickness of *δ* and dive angle measured from horizontal of *θ*, assuming a vertical acoustic beam (Fig 1). Horizontal swath coverage is therefore inversely proportional to dive angle.

$$l=\frac{\left(R-\delta \right)}{\text{tan}\theta }$$ (1)

Despite the inability to measure long targets, gliders have the comparative advantage of carrying echo-sounders beyond the range of those many of the echo-sounders used aboard ships for pelagic surveys, particularly those at higher frequencies. For example, the Simrad EK60 when transmitting at a frequency of 120 kHz is typically used to a range of 500 m[6,20]. The Imagenex ES853 that has been carried by gliders [4] has a range of only 100 m, but with a dive depth of 1000 m, can insonify depths more than double that of its surface-based counterpart. The range bin interval of the ES853 is 0.5 m and beam width of 10°, which, when viewed in isolation, compares unfavorably with the less than 0.2 m bin interval and 7.1° beam width achievable with the EK60 120 kHz system [6,20].

A slow-moving vehicle such as a glider is subject to advection by fast-moving currents and eddies, thus strategy optimization may require more flexible piloting, tacking into a current in order to travel upstream. This problem is exacerbated by the evolving nature of the targets, which may be swimming or vertically migrating, thus complicating the capture of accurate measurements of the targets. Significant research has been conducted into routing strategies for piloting gliders in challenging advective conditions [21] and there have been a number of assessments of gliders’ suitability for sampling different parameters [4,5,22,23]. There are, however, no existing discussions of glider-based acoustic sampling performance with respect to accuracy of measurement under different target and environmental conditions.

New technologies such as multi-beam sonar and wideband acoustics have been simulated to assess their respective abilities to resolve marine targets and to understand their errors and limitations [24–26]. These simulations typically consider the backscattering strength of the targets with respect to the acoustic properties, with results varying by target orientation, use and frequency in use. Target orientations and densities are convoluted in the acoustic data [27–29], thus considerable research effort has been expended in target identification [30–32] and target strength modeling [33–37]. The apparent shape, and particularly the length of targets such as shoals and swarms have also been modeled for sensitivity to inter-ping distance, beam width and target depth for different instruments [38,39], in some cases with post-processing calculations suggested to correct the geometry of the targets [38], caveated by limitations of beam width and range, and are not appropriate for complex targets [40]. With the arrival of new, dynamic platforms, and their anticipated use for acoustic surveys, it would seem an opportune time to assess their relative strengths and weaknesses in resolving targets and accurately recording acoustic backscatter.

Presented here is a comparison of simulations of the error in acoustic sampling of a glider and ship, with parameters typical of previous surveys. The simulations use the modeled collection of normalized backscattering coefficient in linear space (rather than dB in logarithmic space), as targets are idealized scatters with no angular dependence or reverberation. The simulation code is made publicly available and is extensible to include factors such as target angular dependence. Here, the simulation is used to explore the difference in sample volume coverage and overlap, difference in resolving ability, both of complex targets and target spacing, and sensitivity of the platforms to relative motion.

## Materials and methods

### The sampling simulator

#### The development environment

The sampling performance of sea-going platforms was simulated using a package written with Mathworks Matlab, having a two-dimensional array to represent a vertical slice through the ocean with axes representing distance along-track and depth. The simulator then envisages how this space might be sampled given the following considerations:

- Vehicle velocity and dive angle (if any)
- Horizontal and vertical offset to the transducer, such as from the draft of a ship or drop keel
- Transducer orientation
- Acoustic beam range, bin interval and blanking distance
- Ping interval
- 3 dB beam angle
- Beam-pattern

A ‘target’ can then then be positioned anywhere within this space and given horizontal and vertical velocities. The simulation grid resolution is user configurable and the code and example parameters are available for download or repository cloning at https://bitbucket.org/account/user/antarctic_gateway_partnership/projects/GS.

The simulator calculates a vehicle path to the extents of the space (e.g. maximum depth and distance). A theoretical beam is generated based on provided echo-sounder characteristics and orientation. At each sample interval, the beam is positioned relative to the vehicle’s calculated location within the space and used to mask target data in the array, to the accuracy of the simulator space resolution, which is user-configurable. Masked data are averaged by beam bin and added to a collection array for output, analogous to acoustic range bin integration. A record of the number of times each cell has been ‘touched’ by the beam is also kept.

Target data and simulated sampling is normalized in the linear domain. The logarithmic unit dB is not used in the simulation, except in defining the beam pattern (discussed below). As the focus of the simulator is the sampling behavior with respect to physical space, rather than their manifestation in acoustics, the linear domain is useful for direct consideration of error. The modeling of echo-sounder performance and target backscattering strength is a maturing field, the results of which can be applied here rather than recreated.

### Beam definition

**Fig 2:**
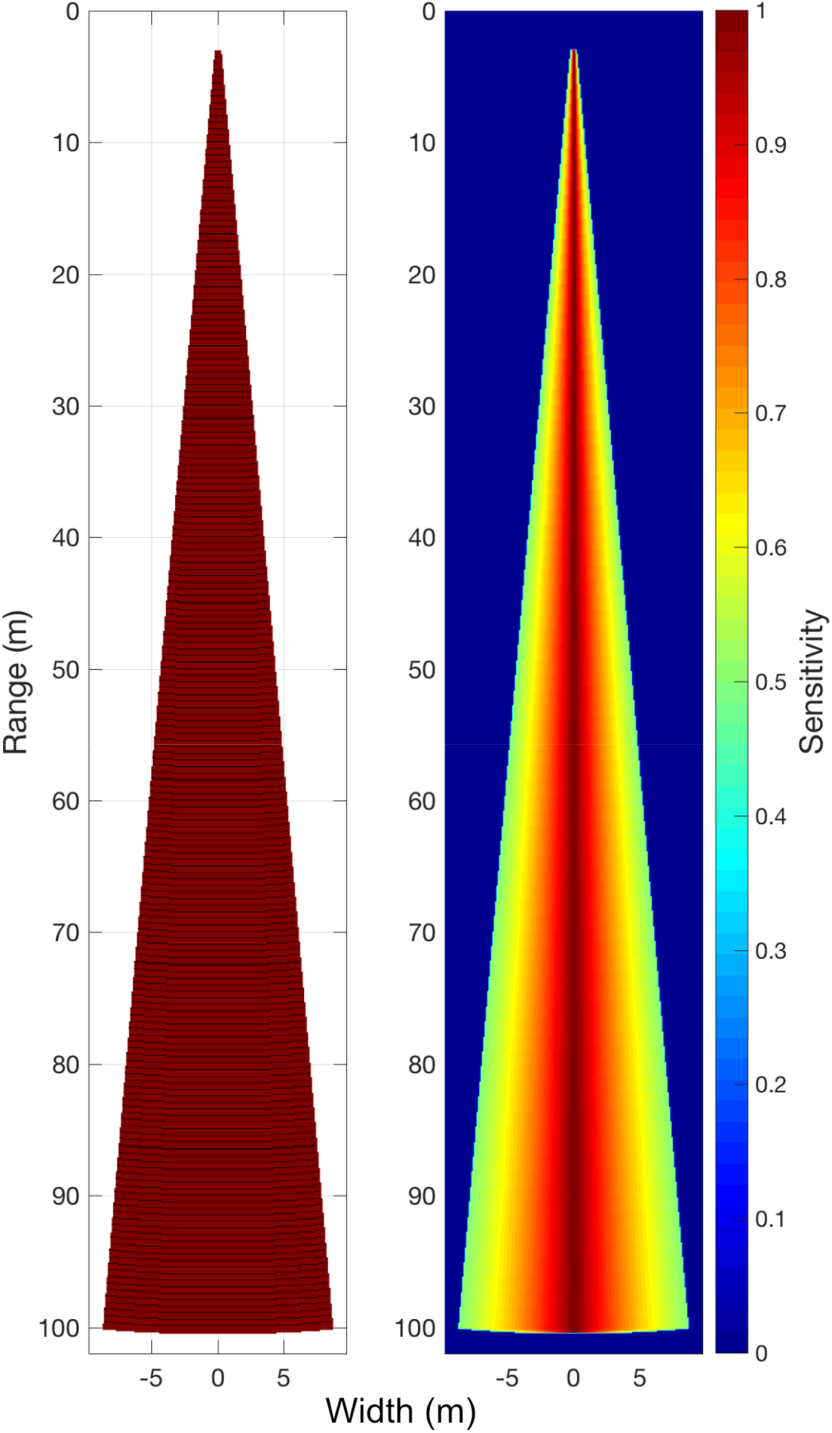
(a) The spatial form of the sampling beam is shown with `gridded bin ranges for the 10° glider beam. (b) The 3° beam definition applied in linear space, normalized and used as a multiplier for overlaid model space, to simulate the varying sensitivity in the sampling volume due to acoustic beam pattern.

The basic beam mask is created in the simulator from the range, bin and angle information that is provided at the input. An empty array with a resolution matching the simulation space and dimensions corresponding to the widest spread and furthest range of the beam, plus a 2% margin, is created. The distance and angle of each cell to point representing the transducer face are calculated with angles less than and equal to the half the beam angle replaced with 1, resulting in a shape that matches the idealized echo-sounder. The range of each cell is assumed to be the midpoint of each sampling volume. The beam ranges are related to sampling bins by identifying the array cells that fall within the extents of each sample volume range (Fig 2a). The beam mask binary matrix is further used to build arrays for testing the sensitivity of the sampling arrangement to different acoustic phenomena. This simulation makes use of the variable response due to beam pattern. The code available also allows for the consideration of incident angle on the data collection, but as this is target-specific it is not considered here.

### Beam pattern effects

The beam pattern can be simulated to be equally sensitive across the cone, or in the form of the idealized -3 dB beam [41]. The 3 dB beam is described in the simulation as a vector of equal-increments of power from -3 to 0 to -3, to the edges of each beam range, before conversion to sensitivity in linear space using Eq 2, where *p* is power. Application to each row of the beam mask produces a beam pattern bias array, from 0.5012 at the edges to 1 in the center (Fig 2b). Each result discussed here makes use of the 3 dB beam pattern.

$$sensitivity=10^{\frac{p}{10}}$$ (2)

### Target definition

Targets are optional in the simulation. They are provided as a 2-dimensional array (values between 0 and 1) and subsequently given a horizontal and vertical scale, seed position in the simulation space and, if desired, a vertical and horizontal velocity. At each ping interval, the sampling mask is repositioned over the space, and the overlap multiplied. A sampling mask copies the overlap with fidelity, while a second mask applies the beam sensitivity to the target. The copied sample is then averaged by each range bin, producing a vector, reflecting the mean volume backscattering data collected by real-world echo-sounders. Each vector is representative of a single ping and is stored alongside time and position.

### Simulation scenarios

#### Platforms

The range of potential scenarios is enormous and those presented here are focused on exploring the glider as a sampling platform, with reference to current ship-based platforms. The following scenarios were simulated using the package described above. The sampling of the space was simulated with two different platforms; a research vessel with a typical survey echo-sounder, and a glider with a compact echo-sounder during downcast (Table 1). Both platforms assume a downward facing, single-beam echo-echo-sounder. Survey parameters were based on previous surveys by ships [20] and gliders [4]. These values represent individual surveys and other uses may vary considerably by echo-sounder available, target species and so on. Additional platforms were simulated to test the sensitivity of the sampling efficacy to changes in platform or echo-sounder design.

**Table 1:** Definition of the sampling platforms and associated echo-sounders.

	Ship	Glider
Horizontal velocity (m s-1)	5 (~ 10 kn)	0.25
Dive angle (°)	0	26
Vertical offset (m)	-7	0
Beam angle (°)	7.1	10
Beam range (m)	500	100
Bin interval (m)	0.1	0.5
Sample interval (s)	2	1

Two types of simulation were run using these platforms: with targets in the space and without. Simulations without targets were used to evaluate the relative coverage of the space by the acoustic sampling, and the overlap by successive pings on the space. These simulations were run at a horizontal and vertical resolution of 0.5 m, over a distance of 5000 m and to a depth of 1000 m. Simulations involving targets were run at 0.1 m horizontal and vertical resolution, over a distance of 500 m and to a depth of 200 m, or with larger spaces where needed to fully encapsulate the excursion of targets travelling at higher velocities.

#### Apparent target size

The spreading of the acoustic beam with ranges means that successively larger volumes are averaged, with a consequent effect on the appearance and relative intensity of the target. Circular targets of different simulated sizes (1, 2, 5, 10, 15, 20 m) were tested for each platform, and at different depths (10 to 100 m in 10 m increments), yielding a matrix of results. In each case, the targets were placed so that the beam of the platform would cover it; for the glider, this meant setting the dive angle to 0.

### Resolving ability

To test platform resolving ability, 2 circular targets, each of radius 10 m, were positioned with horizontal spacing between them. The spacing was increased by 1 m, over successive simulations from 1 to 20 m spacing for the glider, and the same for the ship with the addition of spacing of 25, 30, 35, 40, 45, 50 m. The additional spacing was required for the further ranges tested for the ship. The spaced targets were also placed at a range of depths, from 2 to 100 m in increments of 2 m for the glider and 10 to 500 m in increments of 10 m for the ship. The maximum depth at which each target could be distinguished separately without overlap was recorded in each case. Glider dive angle was set to 0.

#### Sampling error when the target is moving

**Fig 3:**
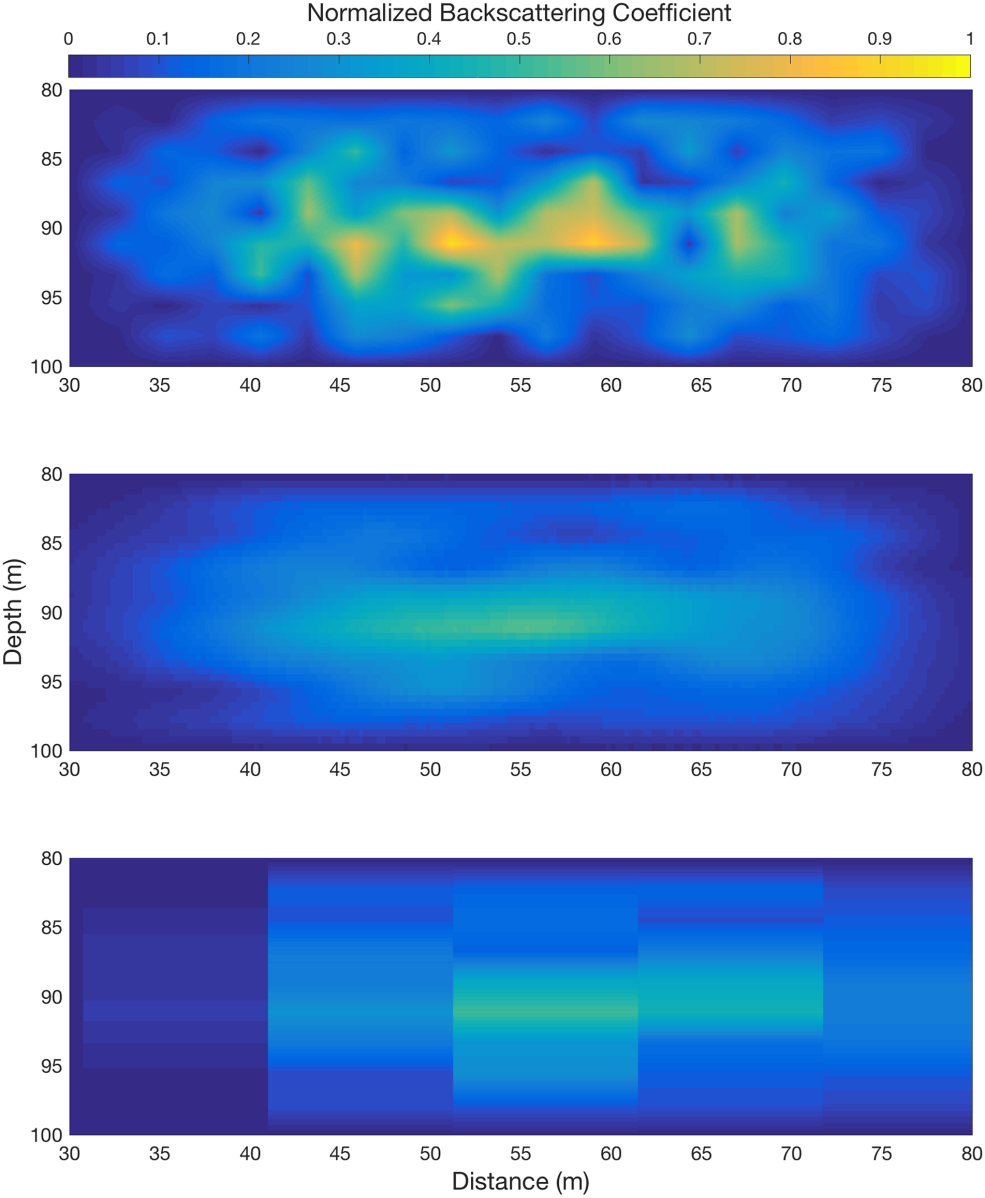
Normalized backscattering coefficient of (a) original generated target and appearance in data collected by (b) a typical glider, and (c) a typical ship.

An abstract target was created (Fig 3a) and the platform sampling was simulated at a wide range of target velocities (-3 to 3 ms-1 in 0.1 m s-1 increments) and target lengths, centered at a depth of 50 m to test sensitivity to target velocity. For each iteration, the percentage error was recorded from difference between the integral of the seeded target array and the sample returned by the platform. In each iteration, the target was placed such that the platform passes over its initial position. The dive angle of the glider was set to 26°.

## Results

### Sample coverage

**Fig 4:**
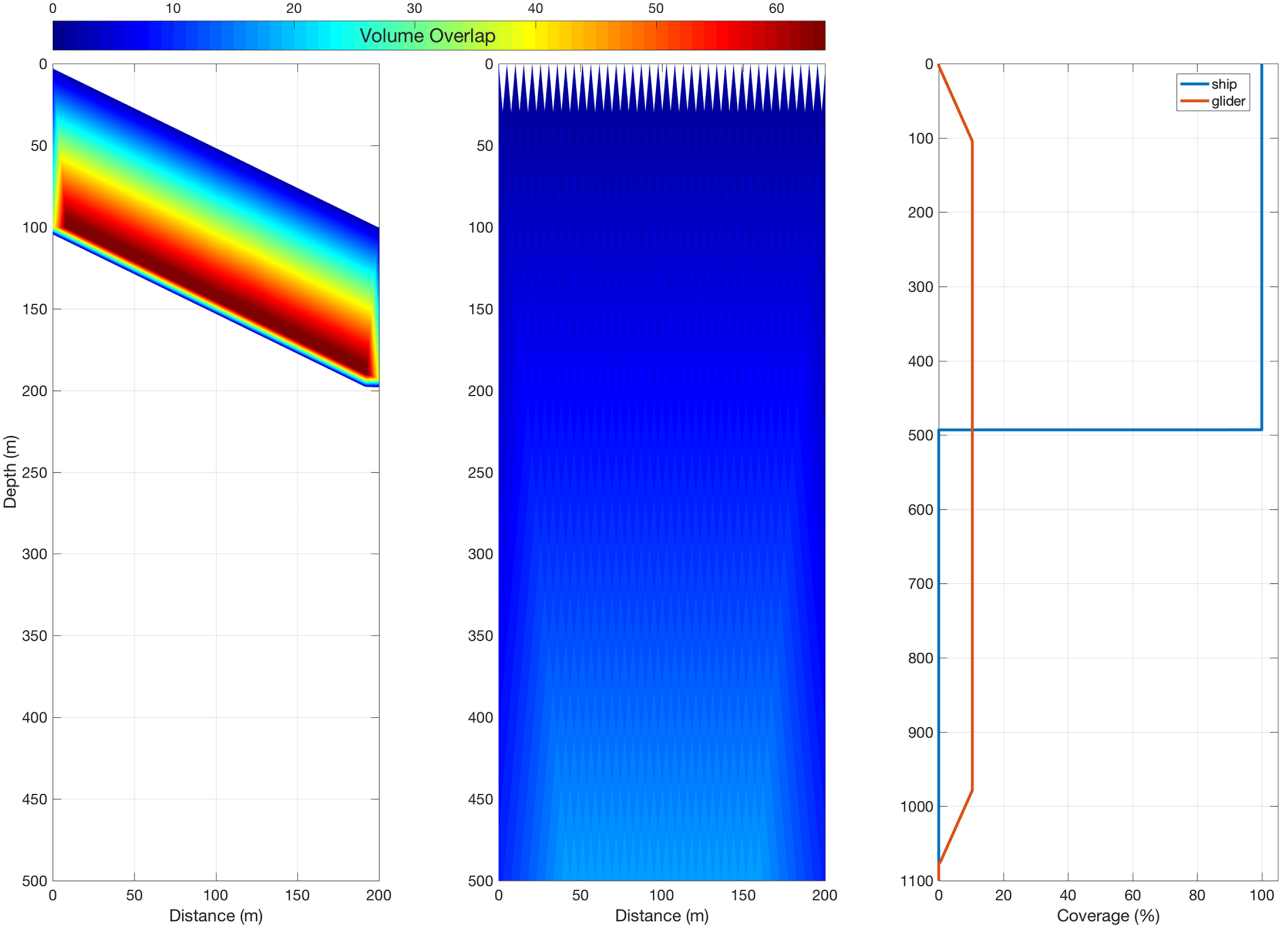
Simulation of insonified volume overlap showing (a) a typical glider with a dive angle of 26°, horizontal velocity of 0.25 ms^-1^ and beam range of 100 m, (b) a typical ship at the surface with a horizontal velocity of 5.14 ms^-1^ (10 knots) and beam range of 500 m. Total horizontal coverage in a 2 km mission by depth is shown for both platforms (c).

A glider and ship, with characteristics described in Table 1, see the world quite differently when considering their 2 dimensional echograms. Despite having an echo-sounder that is coarser in its resolution, the slow, downwards or upwards motion of the vehicle results in a large degree of overlap in the sample volumes (Fig 4a), with up to 67 pings in a volume at the furthest ranges of the beam, the consequence of which is discussed in the next section. The discussion of the simulation assumes a downward-facing echo-sounder, during glider dive. Results are relatable to ascent if echo-sounder orientation is maintained. For simplicity of language only the dive phase is reported. The ship will have considerably less overlap, with fewer than 20 at the maximum simulated range of 500 m, and a maximum of 4 at the maximum range of the glider’s simulated range, 100 m (Fig 2b). The ship could approach this degree of overlap by slowing to the speed of the glider, but this slow progression is generally too slow for a vessel in demand, thus the glider’s volume overlap advantage is a function of economics rather than platform superiority. The horizontal coverage of the glider is 10% at depths greater than the range of the beam, and less than maximum dive depth (Fig 2c). Horizontal coverage is given as a percentage of the total horizontal excursion, thus calculation of fractional horizontal coverage for a dive (angle > 0°), at depths greater than beam range, is simply the maximum horizontal swath (Eq 1) divided by total horizontal excursion, with the angular term cancelled out. Therefore, changing the dive angle will not impact on this coverage. To increase the horizontal coverage in the glider transect the beam range must be increased, which also results in an increase in the length of targets that can be measured. The ship transect will have 100% horizontal coverage, down to the maximum range of the beam. Near the surface there are wedge-shaped gaps in coverage; a function of inter-ping distance and beam angle, extending in this simulation to almost 30 m from the surface.

### Sample resolution

A complex, amorphous target of anisotropic density (Fig 3a) was resolved at a higher resolution by the glider (Fig 3b) than by the ship (Fig 3c), despite the lower resolving power of its echo-sounder. The overlapping bin volumes of the glider, when reoriented in space provide many averages of the same space from different positions, and qualitatively appear to give a more accurate view of the distribution of density within the space that the ship, though features smaller than 5 m are lost in both recreations and the structures are blurred. The simulated data collection is a mean value in each bin, with no gain function applied, as signal attenuation is not simulated. The target was at the same depth, 90 m, for both platforms but the diving of the vehicle brought it to within 50 m of the edge as it passed, but the wider beam angle of the glider increases the ‘attack angle’ [38], causing a smearing of finer features. The absolute error of the measurement by the ship (mean measurement minus ‘real’ value, as a percentage of ‘real’) was -31%, while the glider error was -28%. Decreasing the sampling rate of the glider from 1 to 0.25 Hz had only a marginal impact on the measurement error, increasing it by less than 0.3%. The smoothing of the features in the target by sample volume integration had the effect of both reducing the appearance of small, intense regions, such as those at the center of the ‘real’ data, and also filling in gaps in the target, leading to a loss of structure in the simulated measurements. This smoothing effect, while not a new phenomenon, is a limitation to the coupling of large-scale observations with finer-scale biological processes in the marine environment, and the understanding of features such as swarm structure and small-scale separation.

### Sampling sensitivity

**Fig 5:**
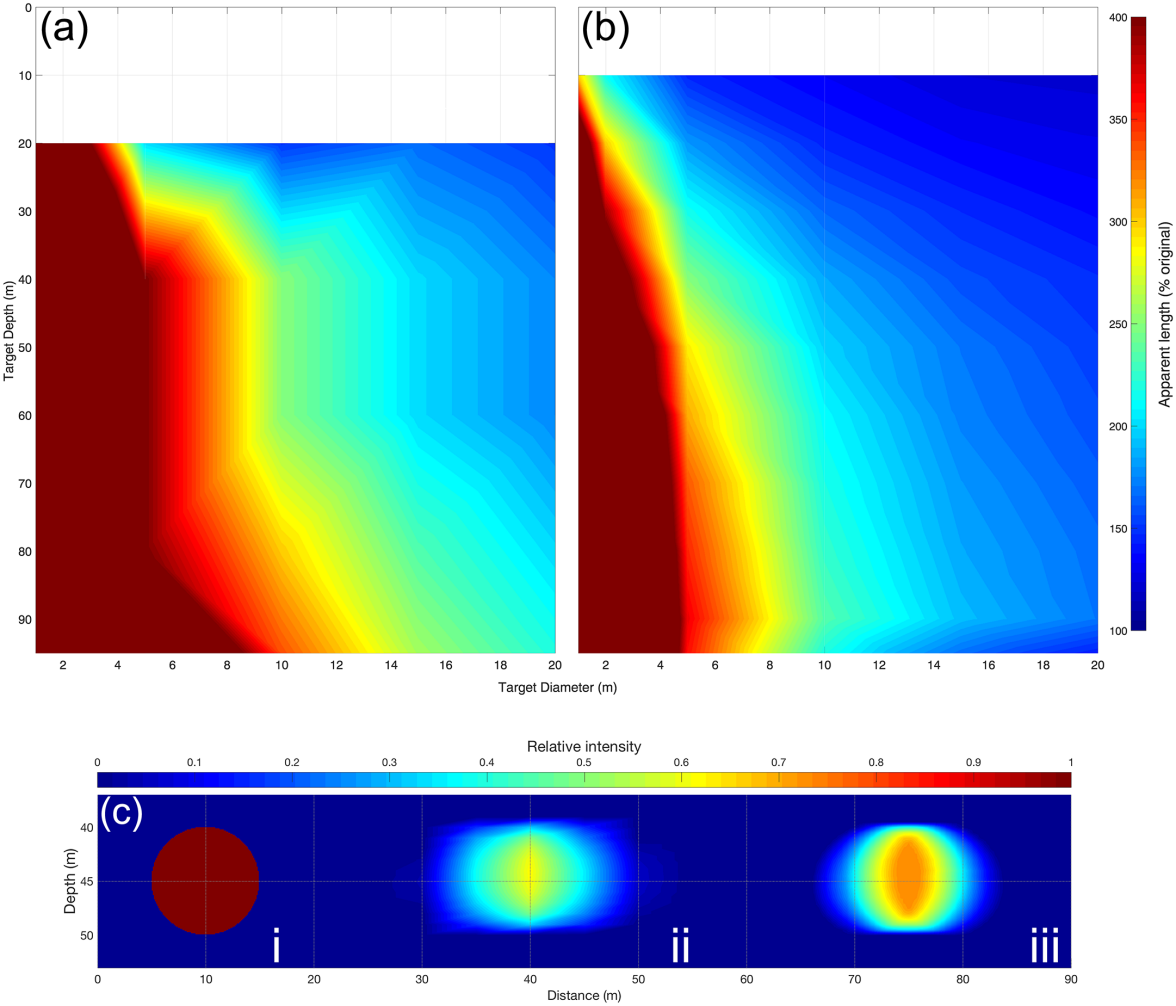
The depth of a circular target is shown against apparent length of a circular target is shown against target diameter, and colored by apparent target length, simulated for (a) a ship and (b) a glider. (c) An example of target appearance is shown for a target of diameter 10 m (i) as it appears in the simulated (ii) ship data, and (iii) glider data.

A homogenous disc, seeded as a target in iterations of simulation with an array of diameters and mean ranges, shows that the target appears longer in the ship transect than in the glider (Fig 5a & 5b). In this instance, the glider was kept at the surface so that the target range is comparable between both platforms, unlike the case of the complex target (Fig 3). Targets with a lengths less than 10 m appear multiple times larger in the ship’s data, reaching an apparent length of 20 m at a range of 25 m, with the error compounded with increased range. The non-linearity in apparent length of target for the ship is a function of the sampling gaps in the upper ranges (Fig 4b). The glider data resolves smaller targets with higher fidelity than the ship (Fig 5b), with the increase in apparent target lengths occurring at a slower rate with respect to range. Targets of lengths less than 5 m appeared considerably longer, except at the closest ranges, <15 m from the glider. The seeded target was reduced in intensity in both simulations (Fig 5c), though the core of the target was preserved with greater fidelity in the glider data.

**Fig 6:**
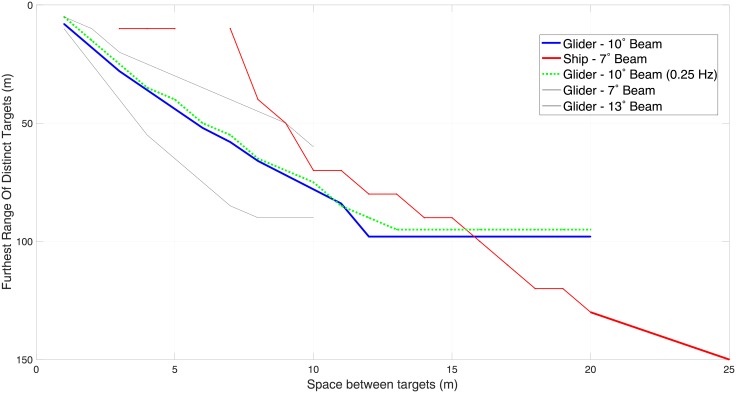
The maximum range at which two targets, each of 10 m diameter and with varying horizontal spacing could be distinguished as separate targets in simulated surveys shown.

Two discs of 10 m diameter, separated by variable horizontal distances were distinguishable as unique targets in the glider at shorter spacing than the ship (Fig 6). The horizontal smearing was reduced by using a threshold value of 0.1, corresponding to a 10 dB difference from the target. In real world applications, the actual threshold value would need to be considered with respect to the target strengths of the features of interest. Adjusting the beam angle of the glider-based echo-sounder demonstrates the sensitivity of the measurement to the speed of the platform. An echo-sounder bourn by the glider with a 7.1° beam angle, as in the ship’s echo-sounder, would be capable of distinguishing hard targets, spaced only 4 m apart, at a range of 50 m. The isotopic hardness of the target used, and particularly its high contrast perimeter bleeds more strongly than a natural, anisotropically dense target, but its use here illustrates the relative ability of the simulated platforms to resolve both small targets and multiple, distinct targets.

### Moving targets

**Fig 7:**
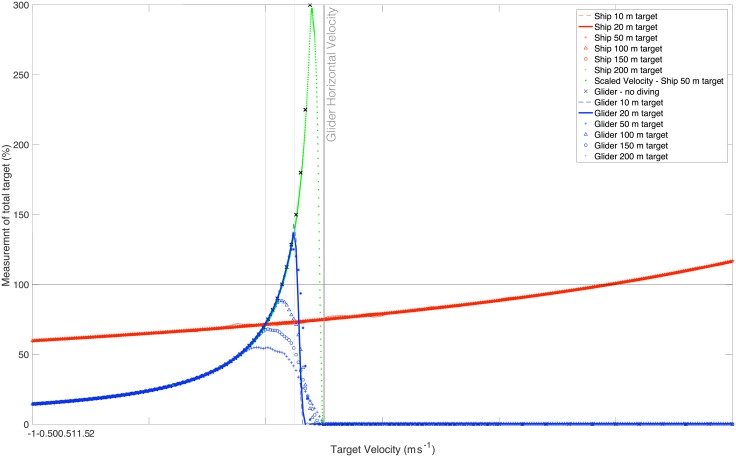
The measurement of a target total normalized backscattering coefficient as a percentage of the actual target is shown over a range of target horizontal velocities, sizes, depths and simulated survey platforms.

The error in measurement of a target is clearly a function of the target’s velocity, with respect to the platform. Targets traveling in the direction of the platform, whether under their own power or advected by current, will appear to have a larger backscattering area than is real, with the limit occurring when the velocity of the target equals or exceeds that of the platform, at which point the target will not be encountered. Conversely, targets traveling in opposition to the platform will appear reduced in size, the effect being asymptotic to an error minimum, which is a function of the beam pattern and the inter-ping distance. The simulation of the sampling of moving targets by the glider and the ship demonstrate that the glider is susceptible to large errors in the observation of moving targets (Fig 7). The size of the target does not influence this error, with the exception of targets that are wider than the swath width (Fig 4). Targets that are to be appropriately resolved at this length would also need to coincide with the arrival and departure of the sampling volumes at target depths, thus any simulation of targets that ‘escape’ the sampling without being fully insonified will result in an under-reporting of backscattering area. Targets of opposing velocity approach an error of 14.5% in measurement. Conversely the glider measures up to 150% the ‘real’ target backscattering coefficient, when the target is traveling at 0.125 ms^-1^ in the same direction, or 50% the glider’s velocity, due to smearing of the target. This velocity limit is a consequence of truncation of the target due to diving behavior, i.e. a target may be aliased over a long distance but the glider will, at a certain point, pass through it, thus terminating the feature. Removing the diving behavior from the glider shows that the percentage measurement, and overestimate, is asymptotic with the platform velocity. This error is a function of relative velocities and thus not limited to the glider, i.e. a target traveling at 50% the velocity of the ship will see the same apparent smearing in the data collected (Fig 7). A platform travelling fractionally faster than the target will reside over the target, thereby grossly overestimating the actual backscattering coefficient. This pattern is also evident in the ship simulations, mapping the error of the glider when velocity is scaled by the to match, a factor of 20.56 for this simulation. The typical ship velocity of more than 5 ms^-1^ means that the errors of this nature likely to occur only in very strong current conditions.

## Discussion

The simulation presented demonstrates the potential for a glider to observe acoustically the pelagic ecosystem at resolutions greater than those typical of research vessels. Resolving finer detail in structure has the potential to improve our understanding of swarming behavior and swarm size with respect to pressures such as fishing and environmental change [6,40,42]. The linear domain is used in these simulation experiments, as the real-world performance of echo-sounders is fraught with considerations of noise, attenuation and target-strength modelling that are unique to specific circumstances [43]. Similarly, the analysis of the simulation outputs is performed on the raw ‘collected’ data, and does not reprocess the data to account for suggested corrections to known errors, such as the target geometry [38] that are applicable in cases of certain target types. A more generalized approach was desired for consideration of gliders as acoustic platforms, rather than an evaluation of specific echo-sounders.

The most obvious reason for the difference between the sampling performance are the diving behavior of the glider, which allows for deep targets to be sampled at close ranges, thus limiting the loss of precision in the spreading acoustic beam. This diving behavior comes, however, at the cost of a limit to the horizontal lengths of the features resolvable. The second major reason for the difference in the sampling of ships and gliders is driven by economics, rather than acoustics, as gliders can afford to travel slowly through the ocean. While a ship could be tasked with travelling slowly and covering 20 km per day, it would likely not be the best use of its time. At these speeds, the ship would not be able to cover the large swaths of ocean needed to make acoustic surveys statistically rigorous. Time aboard a research vessel can cost tens to hundreds of thousands of US dollars per day, though costs are hard to assess as they vary by funding model and platform. Gliders have an upfront cost, typically between $100,000 to $200,000 but come with the risk of loss [44] and the need for associated infrastructure, such as a deployment and recovery vessel.

Thus, a glider seems practical for small-scale observation of individual targets at slower rates and longer durations, while a ship more tuned for the larger, faster, regional scale surveys with less focus on the resolution of individual targets. Research vessel missions and costs also need to be understood in the context of their superiority over gliders for sensor power requirements and complexity, along with the ability to directly recover physical samples. In the case of ecosystem biomass assessment, the sampling of the water column for target identification and target strength modelling is an important step in data processing [20,31,45,46]. The argument can thus be made that gliders should not be thought of as replacing ships for acoustic survey operations but augmenting them, providing new perspectives on spatial and temporal time scales. Multiple gliders, operating in concert, can survey wide swaths of ocean over months at a time, resolving fine targets with the requisite physical sampling undertaken intermittently by research vessels.

Understanding the relative strengths and weaknesses of gliders as an emerging pelagic acoustics platform for biomass assessments means that there is an opportunity for the design of new sensors. Obvious enhancements to glider based echo-sounders, along the lines of advances in ship-based instruments, include the reduction in the beam angle, the use of split-beam transducers and the incorporation of multiple frequencies, of particular importance for target discrimination [34,43,45]. None of these developments will address the fundamental issue of a glider with downwards facing echo-sounder truncating the horizontal sampling due to diving pattern. One approach may be to position the echo-sounders in other orientations, but truncation will remain an issue, with only changes to the geometry. The horizontal scales of glider observation may be increased beyond those represented in this simulation by increasing acoustic range and with the addition of more beam directions. Unless the glider is made to dive to depths less than the maximum effective range of a downward facing echo-sounder there is an inexorable limit to the horizontal scales of observation of contiguous features.

Orientations other than downwards are in use, e.g. in bottom mounted moorings [47,48], where they provide a Eulerian perspective on the pelagic ecosystem. Interpreting acoustic backscatter as pelagic biomass requires the consideration of the target orientation with respect to the beam, and indeed the distribution and diversity of targets in the sample volume [35,37,43]. Calculating an appropriate target strength model for a particular species and beam orientation typically requires the direct sampling, i.e. capture and measurement, of individuals for the assessment of their acoustic properties [30], a task for which gliders, on their own, are ill-suited.

The aliasing of targets due to the slow horizontal speed of the vehicle can cause significant error under survey conditions. While a ship-based survey can be mounted into the current[49], doing so presents two problems for the glider. The glide forward can be met with a strong opposing current that will push the glider backwards. In these conditions, the glider can be commanded to tack into the current, zig-zagging upstream, though this can result in a haphazard, or purposive survey that is inappropriate for abundance estimates using conventional techniques[17]. The second issue is that the glider has typically little or no knowledge of the current dynamics. Depth-averaged currents can be estimated from the flight model of the vehicle and the difference between the GPS positions at the start and end of a dive[50], but this is a bulk estimate and subject to errors in hydrodynamic modeling. One solution to aliasing of targets is for the glider to travel faster into currents using an auxiliary propeller [51] rather than tacking, and to pair acoustic measurements with more granular and precise current estimates using an onboard current profiler [49] and terrain-aided navigation [52], a solution used often by AUVs [53,54]. Greater control of the vehicle in strong currents, and improved localization, allow for the development of adaptive sampling missions and also the calculation of aliasing due to target motion. The accuracy of acoustic target resolution by gliders and the recording of target position is subject to these errors, which grow over time away from the surface GPS, as error accumulate with dead reckoning.

Future large-scale glider surveys are likely to be part of larger sensing networks, with support from research vessels, in this way tying in the scales of observations and qualified target strength models with persistent presence, fine-scale observation and associated ocean profiles of properties such as temperature, salinity, dissolved oxygen and chlorophyll from other sensors [17,55]. The ability to often deploy gliders from small boats in the near shore and to pilot the glider to work area is a significant cost-saving advantage, reducing reliance on support infrastructure like ocean-going ships. Ocean sensing networks incorporating autonomous vehicle have been discussed in various forms for decades [56]. With progress in battery, navigation and sensing technologies, and ancillary infrastructure such as docking, these networks are starting to look feasible [57]. There is an opportunity to reimagine the acoustic sensors we might use for future networks. For example, we might choose to focus less on the horizontal scales measurable with gliders and instead consider them as acoustic profilers, providing rich vertical information beneath points in the ocean, similar to the output of the Argo float network, but with the benefit of active navigation and station-keeping. Such a perspective lends itself well to simulation of sampling error at ocean-scales, as opposed to the single-dive scale presented here.

The degree of synopticity of a survey can be greatly enhanced when the sampling platform’s path and the field’s evolution are carefully considered, e.g. US GLOBEC survey of the copepod *Calanus finmarchicus* on the Grand Banks [58]. Simulations of a "correct" field of data, characteristics of real-world conditions can be sampled with test strategies in a process often called Observing System Simulation Experiments (OSSEs) [55,58], where modeled samples collected by specific strategies are compared for accuracy against the "correct", whole simulated field. Strategic approaches include variation in instrument platform and number, trajectory, speed, and duration. Further advances in understanding how gliders can be tasked with reliably surveying the biomass of the pelagic environment will come with the simulation of the appearance of the targets in an evolving, dynamic space. The inclusion of specific species will include simulated schools, swarms and other structures with known target strengths, angular dependence and behavior e.g. vertical migration, current advection etc. Simulated signal attenuation and signal to noise ratios [59] will help to set the expectations of the platform, while stochastic positioning of targets, evolution of the physical forces affecting both the target and the glider(s) over time, and Monte Carlo simulation of missions will inform the scaling of the glider surveys. All of this is necessary for autonomous networks to ensure that we not introduce more uncertainty than we seek to remove.
